# Meal rich in carbohydrate, but not protein or fat, reveals adverse immunometabolic responses associated with obesity

**DOI:** 10.1186/s12937-016-0219-0

**Published:** 2016-12-01

**Authors:** Ehsan Parvaresh Rizi, Sonia Baig, Muhammad Shabeer, Yvonne Teo, Shao Feng Mok, Tze Ping Loh, Faidon Magkos, Sam Virtue, Antonio Vidal-Puig, E. Shyong Tai, Chin Meng Khoo, Sue-Anne Toh

**Affiliations:** 1 Department of Medicine, Yong Loo Lin School of Medicine, National University of Singapore, 14 Medical Drive, 117599 Singapore, Singapore; 2 Department of Medicine, National University Health System, Singapore, Singapore; 3 Department of Laboratory Medicine, National University Health System, Singapore, Singapore; 4 Department of Physiology, Yong Loo Lin School of Medicine, National University of Singapore, Singapore, Singapore; 5 Singapore Institute of Clinical Sciences (SICS), A*STAR, Singapore, Singapore; 6 University of Cambridge Metabolic Research Laboratories, Cambridge, UK; 7 DUKE-National University of Singapore Medical School, Singapore, Singapore; 8 Perelman School of Medicine, University of Pennsylvania, Pennsylvania, USA

**Keywords:** Mononuclear cells, Inflammation, Gene expression, Diet, Macronutrients

## Abstract

**Background:**

Obesity-related insulin resistance is linked to inflammation. Immunometabolic function differs between lean and obese subjects, but whether macronutrient composition of ingested meals affects these responses is not well known. We examined the effects of a single meal rich in fat, protein, or carbohydrate on immunometabolic responses.

**Methods:**

Nine lean insulin sensitive (LIS) men and 9 obese insulin resistant (OIR) men ingested high-carbohydrate (HC), high-fat (HF) or high-protein (HP) mixed meals in random order. We assessed plasma glucose, insulin, and cytokine responses and cytokine gene expression in circulating mononuclear cells (MNC) at fasting and postprandial states (up to 6-h).

**Results:**

Expression of NF-κB and TNFα genes were greater; whereas that of TGFβ and IL-6 genes were lower, in the OIR compared to the LIS individuals. The differences were significantly greater after the HC meal, but not after the HP or HF meal. Similar results were obtained for plasma concentrations of TNFα and IL-6.

**Conclusions:**

Our findings indicate that a single HC meal has a distinct adverse effect on immunometabolic responses in the OIR individuals. The cumulative effect of such adverse responses to meals rich in carbohydrate may predispose the OIR individuals to a higher risk of cardiovascular disease.

**Electronic supplementary material:**

The online version of this article (doi:10.1186/s12937-016-0219-0) contains supplementary material, which is available to authorized users.

## Background

Obesity-induced insulin resistance is associated with chronic low grade inflammation [1], which is characterized by a progressive increase in pro-inflammatory subsets of immune cells in metabolic tissues, and increased plasma concentrations of various pro-inflammatory cytokines [2–5]. Moreover, circulating mononuclear cells (MNC) in the fasting obese individuals are in a pro-inflammatory state [6].

Nutrient intake poses a transient homeostatic stress, and induces inflammatory responses [7]. A 75-g oral glucose load has been shown to cause an acute inflammatory response, defined by an increase in intra-nuclear nuclear factor κB (NF-κB) binding in MNC [8]. NF-κB is an important inducer of transcription of several inflammatory genes, including genes encoding for cytokines, chemokines and adhesion molecules [9]. A similar inflammatory response to a high-carbohydrate (HC) meal has been reported in MNC in lean individuals [10]. Further, HC meal induces a more prominent and prolonged postprandial oxidative stress and inflammatory response in MNC in obese individuals in comparison to lean individuals [11]. The higher glucose excursion may contribute to such unabated and excessive inflammatory response by acute activation of inflammatory pathways as well as generation of oxidative stress in circulating MNC, macrophages, smooth muscle cells and endothelial cells [12–14]. Such perturbation in regulation of postprandial inflammatory responses, suggested as one of the earliest defects in atherogenesis, may contribute to obese individuals having a higher risk for cardiovascular diseases [14, 15].

A high-fat (HF) meal has also been shown to induce pro-inflammatory responses [16, 17]. In fact, MNC transcriptome better reflects such postprandial pro-inflammatory responses compared to plasma cytokines levels [18]. However, the total amount of fat intake and fatty acid composition of the meal are important determinants of postprandial inflammatory response; and inconsistencies in results of previous studies [19, 20] could be attributed to variations in these factors. Van Djik et al. administered HF meals with different fatty acid composition and showed that differences in the postprandial MNC immune responses between lean, obese non-diabetic, and obese diabetic subjects were dependent on the fatty acid composition of the meal [19]. Further, different inflammatory pathways and responses may be evoked. Manning et al. reported that both low- and high-fat meals had no effect on plasma TNFα and IL-8 concentrations in 15 obese women, but both meals increased IL-6 level [20]. Taken together, the expected inflammatory responses to a high fat meal is likely more complex and nuanced than previously understood; and can vary in different individuals depending on their metabolic status, as well as the composition of fatty acids in the test meal. Hence, there is a need to clarify some of these discrepancies by determining the effect of a standardized HF meal composed of equal proportions of poly-unsaturated (PUFA), mono-unsaturated (MUFA) and saturated fatty acids (SFA).

To our knowledge, there are no studies examining the immunometabolic responses to meals with different macronutrient composition in metabolically distinct, homogeneous cohorts of normoglycaemic obese insulin-resistant (OIR) and lean insulin-sensitive (LIS) individuals. Therefore, we conducted a randomized cross-over study to determine the effects of a single meal rich in fat (PUFA: MUFA: SFA, 1:1:1), protein, or carbohydrate on plasma glucose, insulin, and cytokine responses, and the expression of inflammatory genes in circulating MNC. A better understanding of immunometabolic responses to ingestion of meals differing in macronutrient composition and alterations of such responses in obesity, may facilitate development of novel nutritional strategies in management of obesity and its sequel.

## Methods

### Subjects characteristics

A total of 18 Chinese men aged 21–40 years participated in this randomized, cross-over trial. Demographic data, medical and drug history, and data on lifestyle factors were collected using interviewer-administered questionnaires. Body weight was measured in light clothing to the nearest 0.1 kg using an electronic scale (SECA, Vogel & Halke, Germany). Height was measured without shoes to the nearest 0.1 cm using a wall-mounted stadiometer. Body mass index (BMI) was calculated as weight divided by height squared (kg/m^2^). We used the WHO definition for obesity in Asians [21]. Subjects of two distinct metabolic phenotypes were recruited based on BMI and the Homeostatic Model Assessment-Insulin Resistance (HOMA-IR). This included 9 lean and insulin-sensitive (20 ≤ BMI < 23 kg/m^2^ and HOMA-IR < 1.2) and 9 obese and insulin-resistant (27.5 ≤ BMI < 35 kg/m^2^ and HOMA-IR ≥ 2.5) subjects. We excluded subjects with known first degree family history or personal history of diabetes mellitus, previous or current thyroid disorders, history of malignancy, recent change in the body weight (≥5% during the past 3 months), hospitalization or surgery during the past 6 months, intake of any medication during the past 3 months, daily alcohol consumption > 3 units, present or past history of smoking, and high level of physical activity (>5 h per week).

### Experimental design and meal tests

During the screening visit, height, weight and waist circumference were measured, and fasting blood was obtained for the determination of plasma glucose, serum insulin, electrolytes, non-esterified fatty acid (NEFA) concentrations, and lipid profile. All subjects had a fasting plasma glucose concentration < 5.6 mmol/l. Eligible participants underwent 3 isocaloric liquid mixed meal tolerance tests (MMTTs) differing in macronutrient composition [high carbohydrate (HC), high fat (HF), or high protein (HP)] in random order with 7 days washout in-between each. HC, HF, and HP meals contained 56.4% carbohydrate, 56.5% fat (with a 1:1:1 ratio of SFA, MUFA, and PUFA), and 51.4% protein, respectively (Additional file 1: Table S1). Following a 10-h overnight fast, baseline venous blood samples were collected and subjects were given a 2 510 kJ (600 kcal) liquid mixed meal to consume over 5 min. Fasting (t = 0 min) and postprandial (30, 60, 90, 120, 180, 240, 300, and 360 min) venous blood samples were collected through an intravenous catheter for the determination of plasma glucose and serum insulin concentrations. Separate venous blood samples were collected in EDTA vacutainers at 0, 120, and 360 min for MNC gene expression and measurement of plasma cytokine concentrations.

### MNC RNA isolation, cDNA synthesis and real time reverse transcription-polymerase chain reaction

EDTA-anticoagulated blood samples (9 ml) were layered over 9 ml of Ficoll-paque Plus (GE healthcare, Buckinghamshire, UK) and centrifuged. The MNC layer was harvested and washed with phosphate-buffered saline. Red blood cell lysis was performed according to manufacturer’s instructions (Sigma-Aldrich, St. Louis, MO, USA).

Total RNA from MNC was isolated using the RNeasy Mini Kit (QIAGEN, Netherlands), according to manufacturer’s instructions. Subsequently, 500 ng of total RNA was reverse transcribed using the high capacity cDNA Reverse-Transcription Kit (Applied Biosystems, Waltham, MA, USA), according to manufacturer’s instructions. Real-time reverse transcription-polymerase chain reaction (RT-PCR) was performed using the Vii A 7 Real-Time PCR System (Applied Biosystems). The PCR mix consisted of 2 μL (10 ng) cDNA, 5 μL QuantiFast SYBR Green PCR Master mix (QIAGEN), and 0.1 μL of 100 μmol/L gene-specific primers (AIT Biotech, Singapore). Primer sequences used were chosen based on the sequences available in NCBI nucleotide database and designed using Primer3Plus (http://primer3plus.com/cgi-bin/dev/primer3plus.cgi). The specificity of the PCR products was tested by analysis of the melt curve at the end of the amplification. All samples were run in duplicates and variations in the threshold cycle (CT) between technical replicates were within 10%. All values were normalized to the expression of a housekeeping gene (GAPDH). The expression of GAPDH gene was stable and did not show significant variation across the different time points, meals and phenotypes. The panel of genes examined included IL-6, TNFα, IL-1β, IL-18, IL-8, IL-10, TGFβ, TLR4, MCP-1, and those related to the NFκB complex, i.e., Rel-A (p65 subunit of NFκB), p105 (precursor of p50 subunit of NFκB), and IκB-α and IκB-β (inhibitors of NFκB). We excluded 2 set of samples following the HC meal (1 set from lean subjects and 1 set from obese subjects) due to insufficient or poor quality of RNA.

### Biochemical analysis

Serum glucose and triglyceride concentrations were measured by using enzymatic and colorimetric methods, respectively (AU5800, Beckman Coulter Inc., California, USA). Serum insulin was measured by using a chemiluminescence immunoassay (ADVIA Centaur, Siemens Healthcare Diagnostics, Hamburg, Germany). These analyses were carried out in a laboratory accredited by the College of American Pathologists. NEFA was measured at Mayo Medical Laboratories (Rochester, MN, USA), using an enzymatic colorimetric method (Cobas® 6000, Roche Diagnostics, Indianapolis, USA). Plasma IL-6 (cat no. HS600B) concentrations were measured using Quantikine® high-sensitivity enzyme-linked immunosorbent assay (ELISA) kits (R&D Systems, Minneapolis, MN, USA). Plasma TNFα concentration was measured using an ultrasensitive ELISA kit (cat no. 45-TNFHUU-E01, Alpco Diagnostics, Salem, NH, USA). Intra- and inter-assay coefficient of variations for IL-6 and TNFα were largely within 10%.

### Statistical analysis

Our primary outcome was fold change in transcription of inflammatory genes regulated by NF-κB in MNC from baseline after each meal, as an indicator of NF-κB transcriptional activity and binding. Initial power analysis was based on the postprandial NF-κB expression, in which a sample size of nine subjects per group per test meal was calculated to provide at least 80% power at 5.0% significance level [22]. All statistical analyses were performed using SPSS version 22.0 for Windows (SPSS Inc., Chicago, IL, USA). All variables except NEFA were normally distributed and for NEFA, log transformed values were tested accordingly. All values are presented as means ± standard errors (SEMs) unless stated otherwise. A *P* value of <0.05 was considered statistically significant. Student’s *t*-test was used to compare continuous variables between the two groups at baseline (lean vs. obese).

Fasting insulin (mU/l) × fasting glucose (mmol/l)/22.5 was used to calculate HOMA-IR [23]. Postprandial changes in plasma glucose and insulin concentrations over 6 h were calculated as the incremental area under the curve (iAUC) by using the trapezoid rule. We performed linear mixed modelling to analyse differences among test meals (i.e., HC, HF, or HP) and metabolic phenotypes (i.e., LIS vs. OIR). Test meal and postprandial time were entered as repeated factors in the model, and iAUC (for plasma glucose and serum insulin) or delta values (i.e., changes from baseline; for plasma cytokines and MNC gene expression) were entered as dependent variables; their baseline values were used as covariates in the analyses. If statistical significance was found, post-hoc (Bonferroni) tests were performed to identify differences among test meals and metabolic phenotypes. We also examined if there was any significant interaction between metabolic phenotype and test meal, indicating that differences between lean and obese subjects were test meal-dependent. Pearson’s correlation was performed to examine between and within subjects’ associations between postprandial immunometabolic parameters [24, 25].

## Results

### Subjects

Compared to LIS subjects, OIR subjects were older, had greater BMI and waist circumference, greater HOMA-IR, greater fasting serum insulin and plasma triglyceride concentrations and lower HDL-cholesterol concentration (Table 1). There were no significant differences between the lean and obese groups in fasting plasma glucose, total cholesterol, LDL-cholesterol, and NEFA concentrations, and systolic and diastolic blood pressures (Table 1).

**Table 1:** Subject characteristics at baseline

	LIS subjects	OIR subjects	*P*	Adjusted *P* *
Age (years)	23.22 ± 0.22	28.56 ± 1.40	0.002	
BMI (kg/m^2^)	22.01 ± 0.18	30.15 ± 0.75	<0.001	<0.001
Waist Circumference (cm)	79.94 ± 0.54	100.78 ± 0.98	<0.001	<0.001
Fasting Blood Glucose (mmol/l)	4.33 ± 0.05	4.68 ± 0.12	0.026	0.459
Fasting Serum Insulin (mU/l)	4.31 ± 0.52	21.04 ± 2.27	<0.001	<0.001
Fasting Total Cholesterol (mmol/l)	5.09 ± 0.27	5.28 ± 0.40	0.697	0.791
Fasting Triglyceride (mmol/l)	0.62 ± 0.07	1.98 ± 0.24	<0.001	0.007
Fasting LDL-Cholesterol (mmol/l)	3.09 ± 0.29	3.20 ± 0.35	0.823	0.730
Fasting HDL-Cholesterol (mmol/l)	1.71 ± 0.08	1.19 ± 0.06	0.001	0.005
Fasting NEFA (mmol/l)^a^	0.41 ± 0.04	0.49 ± 0.03	0.037	0.392
Systolic Blood Pressure (mmHg)	110.78 ± 4.07	119.78 ± 2.61	0.084	0.705
Diastolic Blood Pressure (mmHg)	60.56 ± 3.00	71.44 ± 3.18	0.024	0.468
HOMA-IR	0.83 ± 0.10	4.34 ± 0.41	<0.001	<0.001

Data presented as mean ± standard errors of mean. *HOMA-IR* Homeostatic model assessment-insulin resistance, *NEFA* Nonesterified fatty acids, *LIS* Lean insulin sensitive, *OIR* Obese insulin resistant, *P-value adjusted for age. ^a^Analysis was done on log transformed data-sets

### Postprandial insulin and glucose responses

#### Insulin

**Fig. 1:**
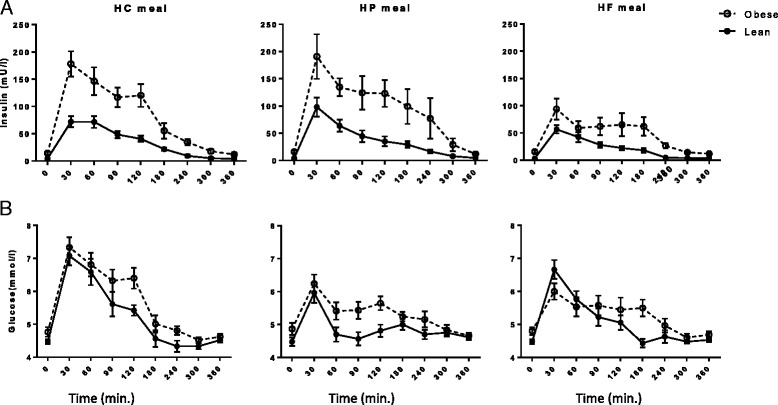
Repeated measurements of (**a**) serum insulin (mU/l) and (**b**) plasma glucose (mmol/l) between lean insulin-sensitive (—, ●) and obese insulin-resistant (−−--, ○) subjects following ingestion of isoenergetic high carbohydrate (HC), high protein (HP), or high fat (HF) liquid mixed meals. Serum insulin iAUC_0–360 min_: *P* = 0.023 for OIR vs. LIS; *P* < 0.001 for between test meals; and *P* < 0.001 for group × test meal. Plasma glucose iAUC_0–360 min_: *P* = 0.673 for OIR vs. LIS; *P* < 0.001 for between test meals; and *P* = 0.157 for group × test meal

OIR subjects had greater incremental postprandial insulin concentration compared to LIS subjects (*P* < 0.05). In particular, among obese subjects, the postprandial insulin response was more robust after HC and HP meals, than HF meal (both *P* < 0.001) (Fig. 1a).

#### Glucose

The iAUC for plasma glucose tended to be greater in the OIR than the LIS group, but this difference did not achieve statistical significance. However, the HC meal induced significantly greater glycemic responses compared to the HP and HF meals (both *P* < 0.001) (Fig. 1b).

### Fasting MNC gene expression and circulating cytokines

Expression of inflammatory genes in MNC and plasma cytokine concentrations in the fasting state are shown in Additional file 1: Table S2. The fasting expression of IκB-α and TLR4 (both *P* < 0.001), and TNFα (*P* < 0.05) genes was significantly lower in the OIR group compared with the LIS group; whereas, IL-6 gene expression was higher (*P* < 0.05). Likewise, plasma IL-6 concentration was greater in OIR than in LIS subjects (*P* < 0.001), but plasma TNFα concentration was not different.

### Postprandial MNC gene expression responses

**Fig. 2:**
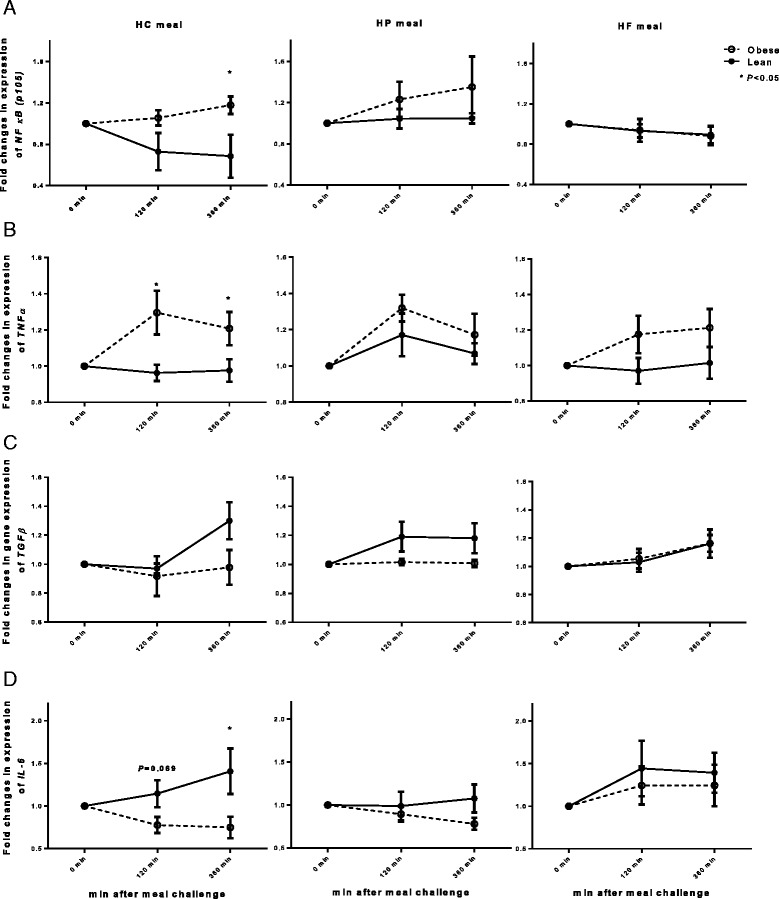
Fold changes from the baseline (fasting) in gene expression of (**a**) p105 (precursor of p50 subunit of NFκB), (**b**) TNFα, (**c**) TGFβ, and (**d**) IL-6 from circulating MNC between lean insulin-sensitive (—, ●) and obese insulin-resistant (−−--, ○) following ingestion of isoenergetic high carbohydrate (HC), high protein (HP), or high fat (HF) liquid mixed meals. NFκB/p105: *P* = 0.012 for group effect; *P* = 0.010 for meal effect; TNFα: *P* = 0.022 for group effect; *P* = 0.325 for meal effect; TGFβ: *P* = 0.016 for group effect, *P* = 0.039 for meal effect; IL-6: *P* = 0.125 for group effect, *P* = 0.007 for meal effect. No significant interactions (group × test meal) were found. Single time point comparisons between two groups, by using unpaired *t* test, are indicated with (*) when significant

Figure 2 shows postprandial changes in inflammatory gene expression (main effects and interactions are presented in Additional file 1: Table S3). The mean fold change in postprandial gene expression for p105 at 6-h and TNFα at both 2-h and 6-h following the HC meal was significantly higher in the OIR compared to the LIS group (*P* < 0.05) (Fig. 2a, b). The mean fold change in postprandial IL-6 gene expression at 2-h (*P* = 0.069) and 6-h (*P* < 0.05) following the HC meal was lower in the OIR compared to the LIS group (Fig. 2d), but this was not the case after the HP or HF meals.

When results for all test meals were collapsed, TGFβ gene expression was lower (*P* for group effect = 0.016) and p105 (*P* for group effect = 0.012) and TNFα (*P* for group effect = 0.022) gene expression was greater in OIR than in LIS subjects. Combined results for all subjects (i.e., for both lean IS and obese, IR subjects) revealed that the HP meal induced a higher expression of p105 (*P* for meal effect = 0.010) and a lower expression of IL-6 (*P* for meal effect = 0.007) compared to the HF or HC meal, while HF meal induced the highest expression of TGF-β (*P* for meal effect = 0.039).

We found positive correlations between TNFα and IL-6 gene expression in both the fasting and the postprandial states (360 min.) in LIS, but not in OIR subjects (Table 2).

**Table 2:** Correlation between fasting and postprandial (at 360 min) MNC gene expression of IL-6 and TNFα in lean, insulin sensitive (LIS) and obese, insulin resistant (OIR) subjects

		LIS Fasting	LIS Postprandial	OIR Fasting	OIR Postprandial
Within subject	Pearson’s *r*	0.60	0.55	0.14	0.13
	*P* value	0.005	0.011	0.566	0.605
Between subject	Pearson’s *r*	0.88	0.51	0.23	−0.10
	*P* value	0.002	0.161	0.548	0.800

We did not find any statistically significant effects of the test meal, differences between lean and obese groups, and phenotype × test meal interactions in MNC gene expression of IL-1β, IL-18, IL-10, IL-8, TLR4, MCP-1, RelA, IκB-α and IκB-β.

### Postprandial plasma cytokines responses

**Fig. 3:**
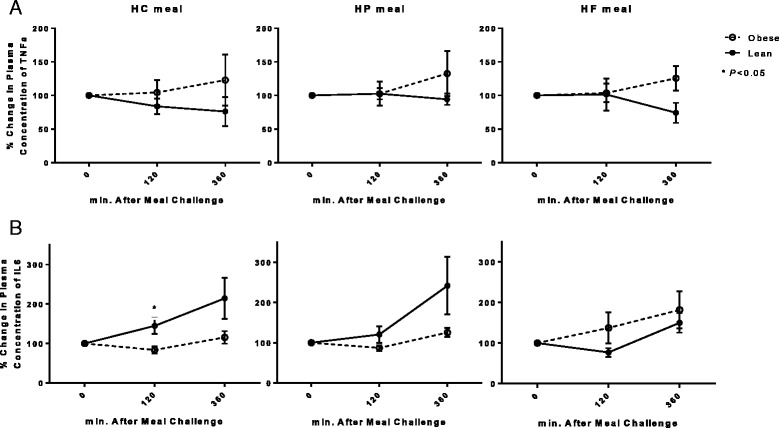
Percentage changes from baseline (fasting) in plasma concentration of (**a**) TNFα, and (**b**) IL-6 between lean insulin-sensitive (—, ●) and obese insulin-resistant (−−--, ○) subjects following ingestion of high carbohydrate (HC), high protein (HP), or high fat (HF) liquid mixed meals. TNFα: *P* = 0.049 for OIR vs. LIS; *P* = 0.645 for between test meals; and *P* = 0.029 for group × test meal. IL-6: *P* = 0.597 for OIR vs. LIS; *P* = 0.727 for between test meals; and *P* = 0.049 for group × test meal

Plasma TNFα and IL-6 concentrations in response to the three test meals are shown in Fig. 3, and summary measures are given in Additional file 1: Table S4. Plasma TNFα concentration changed over time but the change over time differed between the OIR and LIS groups (*P* for time × phenotype =0.09), and it was overall greater in the OIR group than the LIS group (*P* for group effect < 0.05) (Fig. 3a). There was a significant phenotype × test meal interaction (*P* < 0.05), because differences in TNFα between lean and obese subjects were more pronounced after the HC meal than the HP or HF meals.

Plasma IL-6 concentration increased in both groups following all test meals (*P* for time effect < 0.001) (Fig. 3b). This increase was more marked in LIS than in OIR subjects after the HC (*P* < 0.05 at 2-h) meal.

## Discussion

In this study, we compared postprandial metabolic and inflammatory responses in plasma and MNC after ingestion of HC, HF and HP meals. Our study population consisted of a metabolically distinct, homogeneous cohort (for ethnicity and gender) of obese insulin resistant (OIR) and lean insulin sensitive (LIS) individuals. We found that in the OIR group, the HC meal induced high insulin and glucose responses, whereas the HP meal induced a high insulinemic response with a significantly lower glucose response compared to the other test meals. In the LIS group, the HC meal induced a higher glycaemic response but there were no differences in insulin responses among the three test meals.

The significantly higher plasma insulin levels in the OIR compared to the LIS group after the HC meal (despite the similarly high glycaemic responses) reflects peripheral tissue insulin resistance in the OIR subjects, who require more insulin than LIS subjects to maintain the same glucose tolerance. However, after the HP meal, we observed a similar trend for increased insulin response in OIR, but not LIS subjects in the absence of major increases in postprandial glucose levels. The HP meal in this study mainly consisted of whey protein, which has been shown to have an insulinogenic effect with only mild changes in glycaemia [26].

The OIR individuals exhibited a more marked inflammatory response in plasma and MNC following all three test meals compared to the LIS individuals. In agreement with our findings, Patel et al. showed that a single HC meal induced a significantly more prolonged and greater oxidative and inflammatory stress in obese compared to lean individuals [11]. During hyperglycemia, increased synthesis of diacylglycerol (DAG) in endothelial cells, smooth muscle cells, monocytes and macrophages leads to activation of protein kinase C (PKC) pathway. Furthermore, advanced glycation end products (AGEs) are formed, taken up by the aforementioned cells and results in activation of mitogen-activated protein kinase (MAPK) pathway and subsequently NF-κB pathway. This finally culminates in production of cytokines, complement activation and generation of oxidative stress [12–14].

However, we observed that differences between the lean and obese group were dependent on the macronutrient composition of the meal, being greater after the HC meal, but not after the HP or HF meal. We also found that the HC meal upregulated NFκB gene expression in the MNC of the OIR but not the LIS individuals. Upregulation of NFκB gene expression was not significant after the HP and the HF meal, as previously shown by other investigators [19, 27].

Indeed, upregulation of inflammatory signalling pathways following administration of HFHC meal have been observed previously [11, 22, 28]. However, these observations have not been entirely consistent across all studies. More recently, van Dijk et al. reported that pro-inflammatory responses could not be detected in MNC in lean, obese, and obese diabetic individuals, following three different HF meals which differed only in fatty acid composition [19]. It is worth noting that the total amount of fat intake and fatty acid composition of the meal can be important determinants of postprandial inflammatory response. Variations in these factors could have led to the discrepant results between previous studies, the study by van Dijk et al., and the current study. The caloric content of the HF meal in our study is only 2500 kJ, and represents a milder stimulant for induction of postprandial inflammatory response compared to previous studies ranging 3800–7500 kJ [11, 19, 22, 28]. In addition, the SFA proportion was much higher in the aforementioned studies ranging 17-20 g, as compared to our study which was only 12 g. The equal proportions of SFA, PUFA and MUFA in the HF meal could have contributed to the more modest or minimal changes observed in our study, since only SFA and n-6 PUFA in particular, have been reported to exert pro-inflammatory responses [29].

Overall, our findings indicate that test meal composition is an important parameter that can help reveal obesity-related alterations in immune function in relationship to metabolic function. NFκB is a pro-inflammatory transcription factor, and its increase in the nucleus characterises inflammation at the cellular level. In this study, we showed that expression of pro-inflammatory genes regulated by NFκB, such as TNFα, was increased in obese subjects, indicating increased NFκB transcriptional activity and binding. TNFα gene expression exhibited similar meal-specific responses in the OIR and LIS group. In addition, we showed that anti-inflammatory genes, such as TGFβ, were downregulated in the OIR group compared to the LIS group. Although we cannot make any causal inferences, our observations support the idea that obesity-related insulin resistance is associated with inflammation, which can be reliably assessed by evaluating MNC gene expression. Importantly, however, manifestation of altered immunometabolic function in obesity varies in response to ingestion of meals with different macronutrient composition.

Mohanty and colleagues showed that a single HP meal (rich in casein) increases generation of reactive oxygen species in MNC and neutrophils in healthy overweight individuals [30]. To our knowledge, there is no previous study that compared the inflammatory responses in MNC after a single HP meal compared to HC or HF meals. In this study, we showed that a single HP meal induces an overall higher gene expression of p105 compared to the HF or HC meals. Amino acids are thought to be sensed by mammalian target of rapamycin (mTOR) which is an important checkpoint kinase. Activation of the mTOR pathway can induce the unfolded-protein response (UPR) and consequently activate the JUN N-terminal kinase (JNK) which can lead to an increased inflammatory response [7].

We observed that postprandial responses for IL-6 both at the gene expression level and plasma concentrations were greater in the LIS compared to the OIR group, especially following the HC meal. This observation is intriguing and is consistent with a previous study in overweight children using Ensure® enteral supplement as the test meal [31]. The authors showed that fasting plasma IL-6 concentration is positively correlated with insulin sensitivity, suggesting that IL-6 may have a protective rather than a detrimental effect on insulin sensitivity [31]. In fact, in lean subjects, acute IL-6 infusion during hyperinsulinemic-euglycemic conditions enhanced insulin-stimulated glucose disposal [32]. An acute IL-6 exposure has also been shown to inhibit TNFα production [33], suggesting a reciprocal relationship between IL-6 and TNFα in modulating insulin sensitivity. We found positive correlations between TNFα and IL-6 gene expression in both the fasting and the postprandial states in the LIS, but not in the OIR group (Table 2). This may indicate tight reciprocal regulation of TNFα and IL-6 that can modulate insulin sensitivity/resistance in response to meal ingestion in LIS individuals, but this regulation is lost in OIR individuals. This notion is supported by the trend for a reduction in postprandial plasma TNFα concentrations in the LIS group but not in the OIR group. It has been suggested that a decrease in postprandial plasma TNFα concentration in the lean and overweight but insulin-sensitive individuals [31, 34] minimizes interference with insulin signaling and therefore facilitates insulin-mediated nutrient uptake after a meal consumption. In our experimental setting, this physiologic response was impaired in OIR subjects, which supports the idea that immunometabolic adaptation, i.e., the capacity to handle postprandial nutrient-induced stress, is compromised in the obese, insulin-resistant state.

Strengths of this study include the recruitment of a population of two distinct metabolic phenotypes, which were otherwise homogeneous. Moreover, we administered 3 isocaloric meals in random order which allowed an unbiased comparison between test meals of different macronutrient composition. This study also has some limitations that should be kept in mind when interpreting our results. We only recruited men, so we cannot ascertain whether similar results would be obtained in women. Future studies in women have to be performed taking into account the possible influence of the menstrual cycle on immunometabolic responses. In addition, our sample size was relatively small, but significant differences between groups were still detected, and we believe a larger sample size would only accentuate our results. Still, we only assessed the responses to a single meal, so it is not known if our results would be affected by long-term consumption of diets rich in different macronutrients.

## Conclusion

In conclusion, we found that obesity-associated alterations in the immunometabolic response to a mixed meal depend on macronutrient composition, being more pronounced after a HC meal but not after a HP or a HF meal. These changes occurred alongside changes in NFκB gene expression in MNC. Our findings provide new insights into the complex interactions between inflammatory processes and underlying metabolic abnormalities in obesity, and highlight the potential importance of nutritional strategies that could be used to prevent unfavourable immunometabolic responses.

## Additional file

Additional file 1: Table S1. Macronutrient composition of the 3 different liquid mixed meals. **Table S2.** Baseline MNC gene expression and plasma cytokine concentrations. **Table S3.** Fold changes in MNC gene expression at 2 h and 6 h in lean and obese subjects after consuming 3 meals with different macronutrient composition. **Table S4.** Changes in plasma concentration of cytokines at 2 h and 6 h in lean and obese subjects after consuming 3 meals with different macronutrient composition. **Table S5.** Fasting and postprandial plasma concentration of cytokines in lean and obese subjects after consuming 3 meals with different macronutrient composition. (DOCX 57 kb)
