# Supplementary material for: Meal rich in carbohydrate, but not protein or fat, reveals adverse immunometabolic responses associated with obesity
Source: Nutr J. 2016 Dec 1;15:100. doi: 10.1186/s12937-016-0219-0 (PMC5131405; doi:10.1186/s12937-016-0219-0)
Supplement: Additional file 1: Table S1. — Macronutrient composition of the 3 different liquid mixed meals. Table S2. Baseline MNC gene expression and plasma cytokine concentrations. Table S3. Fold changes in MNC gene expression at 2 h and 6 h in lean and obese subjects after consuming 3 meals with different macronutrient composition. Table S4. Changes in plasma concentration of cytokines at 2 h and 6 h in lean and obese subjects after consuming 3 meals with different macronutrient composition. Table S5. Fasting and postprandial plasma concentration of cytokines in lean and obese subjects after consuming 3 meals with different macronutrient composition. (DOCX 57 kb) [file 12937_2016_219_MOESM1_ESM.docx]

**Supplementary Table 1.** Macronutrient composition of the 3 different liquid mixed meals

|  |  | **Amount**  **(g)** | **Energy**  **(Kcal)** | **Carb.**  **(g)** | **Protein**  **(g)** | **Fat**  **(g)** | **MUFA**  **(g)** | **PUFA**  **(g)** | **SFA**  **(g)** |
| --- | --- | --- | --- | --- | --- | --- | --- | --- | --- |
| **HF Meal** | *Ensure Plus^®^* | 260.0 | 366.60 | 51.69 | 13.00 | 11.70 | 2.85 | 6.90 | 1.48 |
|  | Butter | 11.0 | 78.87 | 0.01 | 0.09 | 8.80 | 2.31 | 0.33 | 5.61 |
|  | Diary Cream Heavy Whipping | 11.0 | 37.95 | 0.31 | 0.22 | 4.07 | 1.10 | 0.15 | 2.53 |
|  | Peanut Oil | 13.0 | 114.92 | 0.00 | 0.00 | 13.00 | 5.98 | 4.16 | 2.18 |
|  | Total |  | **598.34** | 52.00 | 13.31 | 37.57 | 12.24 | 11.54 | 11.81 |
|  | % composition |  |  |  |  |  | 32.6 | 30.7 | 31.4 |
|  | % Kcal |  |  | 34.8 | 8.9 | **56.5** |  |  |  |
| **HC Meal** | *Ensure Plus^®^* | 425.0 | 599.25 | 84.49 | 21.25 | 19.13 | 4.65 | 11.28 | 2.42 |
|  | Total |  | **599.25** | 84.49 | 21.25 | 19.13 | 4.65 | 11.28 | 2.42 |
|  | % composition |  |  |  |  |  | 24.3 | 59.0 | 12.7 |
|  | % Kcal |  |  | **56.4** | 14.2 | 28.7 |  |  |  |
| **HP Meal** | *Ensure Plus^®^* | 230.0 | 324.30 | 45.72 | 11.50 | 10.35 | 2.52 | 6.11 | 1.31 |
|  | *Beneprotein^®^* Powder | 77.0 | 274.89 | 0.00 | 65.45 | 0.00 | 0.00 | 0.00 | 0.00 |
|  | Total |  | **599.19** | 45.72 | 76.95 | 10.35 | 2.52 | 6.11 | 1.31 |
|  | % composition |  |  |  |  |  | 24.3 | 59.0 | 12.7 |
|  | % Kcal |  |  | 30.5 | **51.4** | 15.5 |  |  |  |

HC, high carbohydrate, HF, high fat, HP, high protein, MUFA, monounsaturated fatty acids, PUFA, polyunsaturated fatty acids, SFA, saturated fatty acids, *Ensure Plus^®^* (1g=1.41kcal, 0.05g protein, 0.045g fat, 0.1988g carbohydrate, 0.0057g SFA, 0.01095g MUFA, 0.02655g PUFA, 0g fibre) manufactured by *Abbott Nutrition* was used as a benchmark for HC meal; *Beneprotein^®^* (1g powder=3.57kcal, 0g fat, 0g carbohydrate, 0.85g protein, 5mg potassium, 5.7mg calcium, 2mg phosphorus, 0g fibre) is manufactured by *Nestlé Nutrition*.

**Supplementary Table 2.** Baseline MNC gene expression and plasma cytokine concentrations

| MNC Gene Expression (2^-ΔCT^ × 10^-2^) | LIS Group | OIR Group | *P* |  |
| --- | --- | --- | --- | --- |
| IL-1β | 1.61±0.10 | 1.39±0.10 | 0.129 | |
| IL-6 | 0.04±0.01 | 0.06±0.00 | **0.017** | |
| TGFβ | 26.29±1.22 | 24.22±1.18 | 0.229 | |
| **IL-8** | **0.66±0.06** | **0.46±0.10** | **0.136** | |
| IL-10 | 0.04±0.00 | 0.04±0.00 | 0.365 | |
| IL-18 | 2.09±0.08 | 1.95±0.16 | 0.437 | |
| MCP-1 | 0.10±0.01 | 0.09±0.01 | 0.256 | |
| NFκB/p105 | 1.75±0.18 | 2.03±0.13 | 0.226 | |
| Rel-A | 12.64±1.20 | 13.17±0.83 | 0.714 | |
| IκB-α | 8.48±0.36 | 6.56±0.40 | **0.001** | |
| IκB-β | 0.50±0.04 | 0.45±0.03 | 0.371 | |
| TNFα | 1.10±0.05 | 0.94±0.05 | **0.026** | |
| TLR4 | 8.18±0.35 | 5.91±0.52 | **0.001** | |
| **Plasma Cytokine concentration (pg/ml)** |  |  |  | |
| IL-6 | 0.35±0.03 | 0.87±0.09 | **0.001** | |
| TNFα | 1.90±0.33 | 1.99±0.34 | 0.850 | |

Data Presented as mean ± standard errors of mean. TGFβ, transforming growth factor beta, MCP-1, monocyte chemotactic protein 1, NFκB/p105, nuclear factor of kappa light polypeptide gene enhancer in B-cells/p105, Rel-A , nuclear factor-kappa B/p65, IκB-α, nuclear factor Of kappa light polypeptide gene enhancer in B-cells: inhibitor alpha, IκB-β, nuclear factor of kappa light polypeptide gene enhancer in B-cells: inhibitor beta, TNFα, tumor necrosis factor alpha, TLR4, toll-like receptor 4. LIS, lean insulin sensitive, OIR, obese insulin resistant.

**Supplementary Table 3.** Fold changes in MNC gene expression at 2 h and 6 h in lean and obese subjects after consuming 3 meals with different macronutrient composition

|  |  | **HC Meal** | | **HF Meal** | | **HP Meal** | | | **Main Effects** | | | | **Interaction Effects** | | |  |
| --- | --- | --- | --- | --- | --- | --- | --- | --- | --- | --- | --- | --- | --- | --- | --- | --- |
|  |  | Δ 2 h | Δ 6 h | Δ 2 h | Δ 6 h | Δ 2 h | Δ 6 h | Group | | Time | Meal | Group × Meal | | Group × Time | Meal × Time | |
| IL-1β | LIS | 0.77±0.12 | 1.08±0.16 | 0.84±0.06 | 1.14±0.15 | 0.77±0.05 | 0.86±0.05 | 0.918 | | **<0.001** | 0.506 | 0.261 | | 0.318 | **0.026** | |
|  | OIR | 0.79±0.10 | 1.06±0.17 | 0.83±0.11 | 1.49±0.33 | 0.93±0.07 | 1.00±0.05 |  | |  |  |  | |  |  | |
| IL-6 | LIS | 1.14±0.16 | 1.41±0.27 | 1.44±0.33 | 1.39±0.23 | 1.00±0.19 | 1.07±0.16 | 0.125 | | 0.420 | **0.007** | 0.735 | | 0.075 | 0.125 | |
|  | OIR | 0.78±0.09 | 0.75±0.13 | 1.24±0.22 | 1.24±0.24 | 0.89±0.09 | 0.78±0.07 |  | |  |  |  | |  |  | |
| TGFβ | LIS | 0.97±0.03 | 1.30±0.13 | 1.03±0.07 | 1.16±0.10 | 1.10±0.05 | 1.18±0.10 | **0.016** | | **0.001** | **0.039** | 0.648 | | 0.062 | 0.433 | |
|  | OIR | 0.92±0.14 | 0.98±0.12 | 1.05±0.07 | 1.16±0.06 | 1.02±0.02 | 1.01±0.02 |  | |  |  |  | |  |  | |
| **IL-8** | **LIS** | **0.91±0.19** | **0.80±0.36** | **0.82±0.21** | **1.00±0.18** | **0.68±0.17** | **0.52±0.17** | **0.571** | | **0.038** | **0.505** | **0.060** | | **0.822** | **0.939** | |
|  | **OIR** | **0.82±0.17** | **0.46±0.14** | **0.64±0.07** | **0.42±0.13** | **1.07±0.30** | **1.13±0.52** |  | |  |  |  | |  |  | |
| IL-10 | LIS | 1.41±0.31 | 1.77±0.45 | 1.22±0.14 | 1.12±0.09 | 1.38±0.11 | 1.07±0.12 | 0.506 | | **<0.001** | 0.338 | 0.354 | | 0.993 | 0.141 | |
|  | OIR | 1.32±0.15 | 1.18±0.21 | 1.21±0.11 | 1.58±0.19 | 1.45±0.16 | 1.14±0.15 |  | |  |  |  | |  |  | |
| IL-18 | LIS | 0.93±0.07 | 0.94±0.04 | 0.96±0.05 | 0.98±0.05 | 0.94±0.09 | 0.90±0.06 | 0.508 | | 0.747 | 0.897 | 0.629 | | 0.391 | 0.611 | |
|  | OIR | 1.04±0.09 | 1.04±0.09 | 0.95±0.08 | 1.03±0.07 | 1.04±0.10 | 0.97±0.11 |  | |  |  |  | |  |  | |
| MCP-1 | LIS | 1.04±0.17 | 1.99±0.54 | 1.03±0.15 | 1.49±0.27 | 1.12±0.09 | 1.74±0.37 | 0.099 | | **<0.001** | 0.789 | 0.565 | | 0.219 | 0.907 | |
|  | OIR | 0.87±0.11 | 1.25±0.17 | 0.91±0.18 | 1.48±0.23 | 1.08±0.19 | 1.41±0.13 |  | |  |  |  | |  |  | |
| NFκB/p105 | LIS | 0.73±0.18 | 0.68±0.21 | 0.93±0.06 | 0.89±0.09 | 1.04±0.11 | 1.05±0.05 | **0.012** | | 0.938 | **0.010** | 0.304 | | 0.105 | 0.188 | |
|  | OIR | 1.05±0.07 | 1.18±0.08 | 0.94±0.11 | 0.88±0.09 | 1.23±0.17 | 1.35±0.30 |  | |  |  |  | |  |  | |
| Rel-A | LIS | 1.05±0.15 | 1.14±0.27 | 1.07±0.12 | 0.99±0.10 | 0.97±0.05 | 0.91±0.06 | 0.221 | | 0.395 | 0.956 | 0.438 | | 0.787 | 0.618 | |
|  | OIR | 1.06±0.08 | 1.03±0.09 | 1.10±0.16 | 0.89±0.06 | 1.09±0.07 | 1.19±0.11 |  | |  |  |  | |  |  | |
| IκB-α | LIS | 0.93±0.07 | 1.04±0.10 | 0.94±0.08 | 0.87±0.07 | 1.01±0.06 | 1.00±0.11 | 0.705 | | 0.853 | 0.903 | 0.275 | | 0.222 | 0.464 | |
|  | OIR | 1.07±0.05 | 1.04±0.10 | 1.14±0.16 | 1.02±0.09 | 0.92±0.07 | 1.06±0.09 |  | |  |  |  | |  |  | |
| IκB-β | LIS | 1.01±0.11 | 1.22±0.22 | 0.99±0.12 | 0.96±0.11 | 1.07±0.07 | 1.11±0.13 | 0.533 | | **0.010** | 0.728 | 0.486 | | 0.541 | 0.510 | |
|  | OIR | 1.06±0.05 | 1.21±0.17 | 1.12±0.12 | 1.15±0.08 | 1.15±0.08 | 1.19±0.11 |  | |  |  |  | |  |  | |
| TNFα | LIS | 0.96±0.04 | 0.97±0.06 | 0.97±0.07 | 1.01±0.09 | 1.18±0.13 | 1.07±0.06 | **0.022** | | **0.001** | 0.325 | 0.249 | | **0.013** | 0.316 | |
|  | OIR | 1.24±0.12 | 1.21±0.08 | 1.17±0.11 | 1.21±0.11 | 1.32±0.07 | 1.17±0.11 |  | |  |  |  | |  |  | |
| TLR4 | LIS | 0.96±0.05 | 1.14±0.07 | 0.89±0.09 | 0.90±0.09 | 1.10±0.11 | 1.18±0.07 | 0.885 | | **0.012** | 0.128 | 0.280 | | 0.370 | 0.156 | |
|  | OIR | 1.06±0.11 | 1.29±0.12 | 1.05±0.15 | 1.11±0.09 | 1.14±0.14 | 1.11±0.11 |  | |  |  |  | |  |  | |

Data Presented as mean ± standard errors of mean. LIS, lean insulin sensitive, OIR, obese insulin resistant, HC, high carbohydrate, HF, high fat, HP, high protein. TGF-β, transforming growth factor beta, MCP1, monocyte chemotactic protein 1, NFκB/p105, nuclear factor of kappa light polypeptide gene enhancer in B-cells/p105, RelA, nuclear factor-kappa B/p65, IκB-α, nuclear factor of kappa light polypeptide gene enhancer in B-cells: inhibitor alpha, IκB-β, nuclear factor of kappa light polypeptide gene enhancer in B-cells: inhibitor beta, TNFα, tumor necrosis factor alpha, TLR4, toll-like receptor 4.

**Supplementary Table 4.** Changes in plasma concentration of cytokines at 2 h and 6 h in lean and obese subjects after consuming 3 meals with different macronutrient composition

|  |  | **HC Meal** | | **HF Meal** | | **HP Meal** | | **Main Effects** | | | **Interaction Effects** | | | |  |
| --- | --- | --- | --- | --- | --- | --- | --- | --- | --- | --- | --- | --- | --- | --- | --- |
|  |  | Δ 2 h | Δ 6 h | Δ 2 h | Δ 6 h | Δ 2 h | Δ 6 h | Group | Time | Meal | | Group × Meal | Group × Time | Meal × Time | |
| IL-6 | LIS | 0.20±0.05 | 0.49±0.22 | -0.09±0.06 | 0.23±0.11 | 0.05±0.06 | 0.38±0.16 | 0.597 | **<0.001** | 0.727 | | **0.049** | 0.142 | 0.984 | |
|  | OIR | -0.18±0.13 | 0.09±0.15 | 0.06±0.07 | 0.28±0.11 | -0.11±0.07 | 0.19±0.08 |  |  |  | |  |  |  | |
| TNFα | LIS | -0.36±0.21 | -1.12±0.66 | -0.08±0.15 | -0.21±0.32 | -0.13±0.09 | -0.08±0.26 | **0.049** | 0.380 | 0.645 | | **0.029** | 0.099 | 0.899 | |
|  | OIR | 0.15±0.30 | 0.36±0.26 | 0.15±0.28 | 0.07±0.34 | -0.34±0.21 | -0.15±0.19 |  |  |  | |  |  |  | |

Data Presented as mean ± standard errors of mean. LIS, lean insulin sensitive; OIR, obese insulin resistant; HC, high carbohydrate; HF, high fat; HP, high protein; TNFα, tumor necrosis factor alpha.

**Supplementary Table 5.** Fasting and postprandial plasma concentration of cytokines in lean and obese subjects after consuming 3 meals with different macronutrient composition

|  |  |  | **HC Meal** | |  | **HF Meal** | |  | | **HP Meal** | |
| --- | --- | --- | --- | --- | --- | --- | --- | --- | --- | --- | --- |
|  |  | 0 min. | 120 min. | 360 min. | 0 min. | 120 min. | 360 min. | 0 min. | 120 min. | | 360 min. |
| IL-6  (pg/ml) | LIS | 0.34±0.03 | 0.52±0.06 | 0.82±0.21 | 0.42±0.09 | 0.37±0.08 | 0.75±0.15 | 0.30±0.05 | 0.35±0.07 | | 0.68±0.17 |
|  | OIR | 1.07±0.20 | 0.98±0.20 | 1.33±0.28 | 0.70±0.13 | 0.76±0.11 | 0.99±0.12 | 0.87±0.13 | 0.76±0.14 | | 1.06±0.17 |
| TNFα  (pg/ml) | LIS | 2.16±0.72 | 1.80±0.70 | 1.04±0.43 | 2.25±0.50 | 2.17±0.51 | 2.03±0.46 | 1.31±0.48 | 1.13±0.56 | | 1.23±0.69 |
|  | OIR | 2.03±0.53 | 2.18±0.67 | 2.39±0.64 | 1.63±0.69 | 1.77±0.70 | 1.96±0.67 | 2.32±0.59 | 1.98±0.49 | | 2.44±0.42 |

Data Presented as mean ± standard errors of mean. LIS, lean insulin sensitive; OIR, obese insulin resistant; HC, high carbohydrate; HF, high fat; HP, high protein; TNFα, tumor necrosis factor alpha.
